# MrsRF: an efficient MapReduce algorithm for analyzing large collections of evolutionary trees

**DOI:** 10.1186/1471-2105-11-S1-S15

**Published:** 2010-01-18

**Authors:** Suzanne J Matthews, Tiffani L Williams

**Affiliations:** 1 Department of Computer Science and Engineering, Texas A&M University, College Station, TX

## Abstract

**Background:**

MapReduce is a parallel framework that has been used effectively to design large-scale parallel applications for large computing clusters. In this paper, we evaluate the viability of the MapReduce framework for designing phylogenetic applications. The problem of interest is generating the all-to-all Robinson-Foulds distance matrix, which has many applications for visualizing and clustering large collections of evolutionary trees. We introduce MrsRF (*MapReduce Speeds up RF*), a multi-core algorithm to generate a *t *× *t *Robinson-Foulds distance matrix between *t *trees using the MapReduce paradigm.

**Results:**

We studied the performance of our MrsRF algorithm on two large biological trees sets consisting of 20,000 trees of 150 taxa each and 33,306 trees of 567 taxa each. Our experiments show that MrsRF is a scalable approach reaching a speedup of over 18 on 32 total cores. Our results also show that achieving top speedup on a multi-core cluster requires different cluster configurations. Finally, we show how to use an RF matrix to summarize collections of phylogenetic trees visually.

**Conclusion:**

Our results show that MapReduce is a promising paradigm for developing multi-core phylogenetic applications. The results also demonstrate that different multi-core configurations must be tested in order to obtain optimum performance. We conclude that RF matrices play a critical role in developing techniques to summarize large collections of trees.

## Background

MapReduce [1] is an exciting new paradigm for designing parallel applications. It was popularized by Google to support the parallel and distributed execution of data intensive applications. To process petabytes of data, Google executes thousands of MapReduce applications per day. There is interest within the bioinformatics community to harness the power of MapReduce to develop parallel applications to process large datasets of genomic data. For example, CloudBurst [2], a MapReduce application for sequence analysis, has recently been released. In this paper, we study whether MapReduce can be used to develop efficient parallel phylogenetic applications for multi-core platforms.

We develop a new algorithm called *MrsRF (MapReduce Speeds up RF) *for computing the all-pairs Robinson-Foulds distance between *t *evolutionary trees on multi-core computing platforms. The RF distance is a popular measure for computing the differences in evolutionary relationships between *t *phylogenetic trees of interest. There are several applications for using RF matrices such as visualizing collections of trees [3,4] and clustering tree collections [5].

The *novelty *of our work centers around using MapReduce in a non-standard way. Typical uses of the MapReduce framework reduce the final output into a smaller representation than the initial input. One of the interesting aspects of the all-pairs RF distance problem is that the output size (a *t *× *t *RF matrix) is much larger than the input size (*t *phylogenetic trees). Under the all-pairs RF problem, we are significantly expanding the data. For *k *total cores, how they are partitioned among the *N *nodes (or physical machines), where each node consists of *c *computing cores, has a significant impact on performance since cores on the same node share resources such as memory bandwidth. For example, with 32 total cores, a 16 nodes by 2 cores (16 × 2) cluster configuration outperforms 8 × 4, 4 × 8, and 32 × 1 configurations in our experiments. Hence, multiple configurations should be tested in order to attain optimum performance on a multi-core platform.

We ran our experiments on 20,000 and 33,306 biological tree collections consisting of 150 and 567 taxa, respectively. MrsRF was implemented using Phoenix [6], a MapReduce implementation for shared memory multi-core platforms, and OpenMPI [7]. Our results show that MrsRF is a promising methodology for parallelizing the all-pairs RF distance problem. In our experiments, MrsRF shows good overall speedup. On 8 cores, MrsRF is over 6 times faster than the best-performing sequential algorithm, which is also MrsRF run on a single core. For 32 cores, it is 18 times faster than the serial version of MrsRF. Speedup resulted from allowing the underlying MapReduce runtime system to schedule communication on the multi-core system, which greatly simplifies MrsRF's implementation.

A common trend in phylogenetics is encapsulating the result into a single consensus tree, where the assumption is the information discarded is less important than the information retained. However, many of the trees may contain elements of the "true" evolutionary tree and their relationships should not be ignored. Hence, we show how to use RF matrices to improve the summarization of a phylogenetic analysis. Overall, our results provide evidence that large computations involving phylogenetic trees can take advantage of the MapReduce framework to design high-performance phylogenetic applications.

## Methods

### MapReduce

MapReduce [1] is a popular parallel model that automates parallel computation largely in the background, making it easier to develop a parallel program. Popularized by Google in 2004, it has since been used for a variety of diverse applications such as distributed sort and grep, Google web indexing, and data processing by large companies such as Amazon, Yahoo! and Facebook. The central features of the MapReduce framework are two functions: map() and reduce(). The map() function produces a set of intermediate key/value pairs. The reduce() function accepts an intermediate key and a set of values and merges them together. Both the map() and reduce() functions are written serially by the programmer. The underlying MapReduce framework takes care of scheduling these functions on the multi-core system.

**Figure 1:**
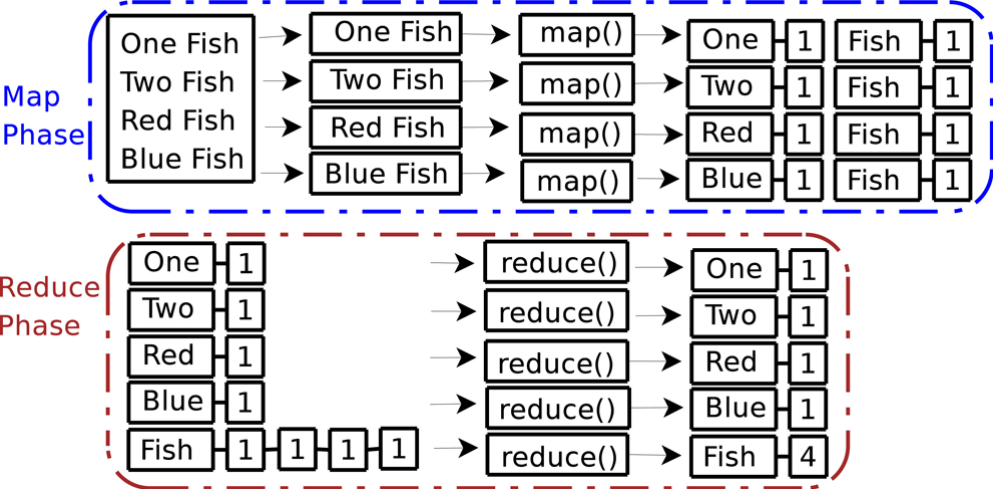
**Word count example using the MapReduce paradigm**.

Figure 1 gives an overview of how the MapReduce paradigm operates in order to count the number of words in a file. Each instance of the map function (or *mapper*) receives one line of input. Each mapper takes its line of input and splits it into words. The mapper then outputs a (key, value) pair of the word and the value 1. Since all the lines are independent from each other, all mappers run in parallel.

As each mapper outputs (key,value) pairs, these pairs are merged to form keys with associated lists of values. In the reduce phase, each instance of the reduce function (or *reducer*) takes as input a key and associated list of values. In Figure 1, the fifth reducer takes in as input the key fish and the associated list 1-1-1-1, as "fish" occurred four times in the input file. Each reducer takes its list of values, sums all of its member's values, and outputs this sum of values with the key.

#### Phoenix: a MapReduce library

The underlying MapReduce framework of MrsRF is based on Phoenix [6], a multi-core MapReduce approach. Phoenix is a threads-based implementation of MapReduce designed specifically for multi-core systems where all computing cores have access to shared memory. It dynamically schedules map and reduce tasks to available compute cores. Hadoop [8] is the most popular framework for developing MapReduce applications. We developed MrsRF implementations in both Phoenix and Hadoop. For our RF matrix application, we were able to achieve significantly better performance using Phoenix. Consider a *N *× *c *multi-core cluster configuration, where *N *represents the number of physical nodes (or machines) of the cluster and *c *is the number of computing cores per node. Each of the cores on a node share access to memory. Phoenix works on a 1 × *c *configuration. We augment Phoenix with OpenMPI in order to use distributed-memory clusters, where *N *≥ 1. Our MrsRF implementation is available publicly from the web [9].

### Robinson-Foulds distance

The Robinson-Foulds (RF) distance [10] is one of the most common methods used to compare the topological differences between two trees. For tree *T *on *n *taxa, removing an edge (or bipartition) splits tree *T *into two independent sets, *S*_1 _and *S*_2_. Each of the *n *taxa belong to either *S*_1 _or *S*_2_. Consider bipartition *B *in tree *T*. We can represent this bipartition as *S*_1_|*S*_2_, where *S*_*i *_contains the names of the taxa in that set. The RF distance computes the topological distance between two trees by comparing their set of bipartitions. Let  define the set of bipartitions found in tree *T*. The RF distance between trees *T*_*i *_and *T*_*j *_is:

In this paper, we develop a multi-core algorithm to compute the *t *× *t *RF matrix for a collection of *t *trees. Entry (*i*, *j*) in the RF matrix represents the RF distance between trees *T*_*i *_and *T*_*j*_. Finally, our results shows the RF rates instead of RF distances. The *RF rate *is obtained by normalizing the RF distance by the number of internal edges and multiplying by 100. For *n *taxa, there are *n *- 3 internal edges in a binary tree. Hence the maximum RF distance between two trees is *n *- 3, which results in an RF rate of 100%. Thus, the RF rate varies between 0% and 100% signifying that the two trees *T*_*i *_and *T*_*j *_are identical and maximally different, respectively.

#### HashRF

Our MrsRF algorithm for multi-core platforms is based on the HashRF algorithm [11,12], a fast, sequential algorithm for computing an all-to-all RF matrix to compare *t *trees on *n *taxa. For a bipartition *B*, HashRF uses a global hash table *H *to store that bipartition along with the identities of the trees (TIDs) that contain that bipartition. HashRF uses two uniform hash functions *h*_1 _and *h*_2_, where the *h*_1 _value represents the hash table location for storing the bipartition *B *and *h*_2 _provides a shortened bipartition identity (BID) for this bipartition. Moreover, for each (*h*_1_(*B*), *h*_2_(*B*)) pair, a list of trees (TIDs) containing bipartition *B *is also stored in the hash table *H*. To compute the RF matrix *M*, each index (*h*_1 _value) of the hash table *H *is visited. At *H *[*h*_1_], each *h*_2 _value (representing a unique bipartition *B*) is visited and its list of tree identities (TIDs) are extracted. For each pair of trees *T*_*i *_and *T*_*j *_in the list of TIDs, entry *M *[*T*_*i*_, *T*_*j*_] is incremented by one to compute a similarity matrix. Once the hash table has been traversed, entry *M *[*i*, *j*] is subtracted from *n *- 3, the maximum RF distance, to produce the RF matrix. The worst-case running time of HashRF is *O*(*nt*^2^).

### MrsRF: Computing a *t *× *t *RF matrix

We introduce *MrsRF (MapReduce Speeds up RF)*, a multi-core all-to-all RF distance matrix algorithm using the MapReduce framework. The design of MrsRF is motivated by the HashRF algorithm. Moreover, in MrsRF, bipartitions are analogous to words in the MapReduce word count example. MrsRF takes as input a tree file containing *t *trees and a *N *× *c *cluster configuration. The number of cluster nodes specifies the number of physical machines that executes the code. The number of cores is the number of CPUs within each node. For serial execution, *N *= 1 and *c *= 1. If instead one wanted to run MrsRF on 2 machines each containing 4 CPUs, the respective *N *and *c *values would be 2 and 4.

**Figure 2:**
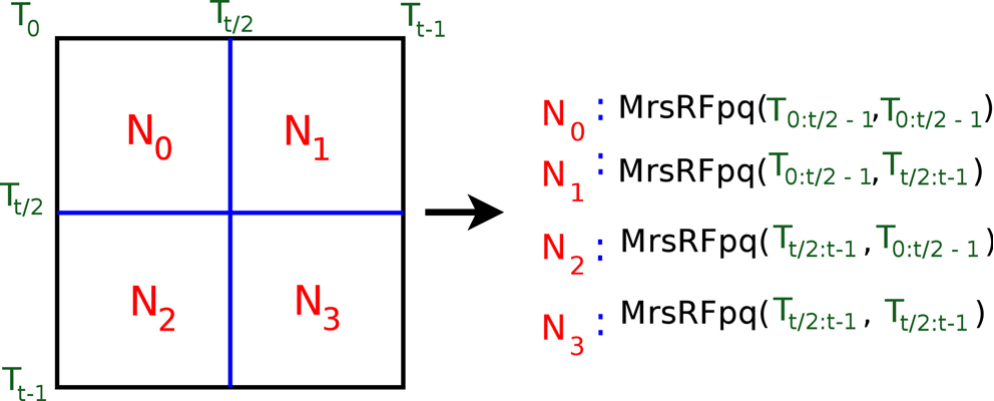
**Global partitioning scheme of the MrsRF algorithm**. Here, *N *= 4. Each of these 4 submatrices will be calculated by a separate instance of MrsRF(*p*, *q*).

There are two main steps to our MrsRF algorithm. First, we organize the *N *nodes into a grid in order to partition the *t *input trees among the nodes. Phoenix, the underlying MapReduce library, automatically distributes the input for a single node amongst its *c *cores. That is, it works for 1 × *c *cluster configurations. As a result, we manually partition the input among the *N *nodes. If *N *is a perfect square, then we assume the nodes are organized into a  grid. If *N *is not a perfect square, let *i *= ⌊⌋. If *N *mod *i *= 0, then we assume a *N*/*i *× *i *grid of nodes. Otherwise, we decrement *i *until it divides *N *evenly. For *N *= 4, the *N *nodes are partitioned into a 2 × 2 grid (see Figure 2). If *N *= 18, we obtain a 6 × 3 grid. The size of the input tree file has no bearing on how the *N *nodes are organized into a grid.

Secondly, once the nodes are organized in a grid using OpenMPI, the MrsRF(*p*, *q*) algorithm is executed on each node to compute a *p *× *q *submatrix. For example, consider node *N*_2 _in Figure 2. Let  and  represent the row and column trees, respectively, in the submatrix. For node *N*_2_,  = {*T*_*t*/2_, ..., *T*_*t*-1_} and  = {*T*_0_, ..., *T*_*t*/2 - 1_}. Hence, the size of node *N*_2_'s submatrix is *p *× *q*, where *p *= || and *q *= ||.

Once each node is finished computing its *p *× *q *submatrix, the final *t *× *t *RF matrix is the concatenation of the *N *submatrices.

### MrsRF(*p*, *q*): Computing a *p *× *q *RF submatrix

The heart of our MrsRF algorithm lies in the subprogram MrsRF(*p*, *q*), which runs independently on each of the *N *nodes. Each node has access to the input file containing the *t *trees and is responsible for retrieving the appropriate trees for its  and  sets. A node knows which trees belongs to its  and  sets based on its identifier within the node grid. In Figure 2,  = {*T*_*t*/2_, ..., *T*_*t*-1_} and  = {*T*_0_, ..., *T*_*t*/2 - 1_} on node *N*_2_. If the number of computing cores on node *N*_2 _is eight, then the trees associated with sets  and  will each be split into four files, yielding a total of eight input files for the eight cores. Under MapReduce, these eight files will be automatically assigned to the cores on node *N*_2_.

Under MrsRF(*p*, *q*) the trees in  are compared to the trees in . If , all trees are compared to each other. Node *N*_*i*_'s submatrix is created in parallel using two MapReduce phases as described below.

#### Phase 1 of the MrsRF(*p*, *q*) algorithm

##### The first map stage

Similarly to HashRF, the first MapReduce phase is responsible for generating the global hash table. That is, every bipartition is given a unique identifier (key). Its values are the tree identities (TIDs) that contain that bipartition. The number of mappers correspond to the number of cores utilized on a particular node. Each mapper sends its trees to HashBase to create a local hash table. HashBase is our name for a modification to HashRF that outputs a hash table from its input trees. Each line from the hash table that is provided to MrsRF(*p*, *q*) from HashBase consists of a bipartition *B*_i_ and the associated list of tree ids that were found to share it. In addition, all the bipartitions that are found are given a marker to denote which input file created it. This is to ensure that bipartitions shared within a tree file are not compared to each other. The bipartition and its list of tree ids form a (key, value) pair, which is emitted as an intermediate for the reduce stage.

**Figure 3:**
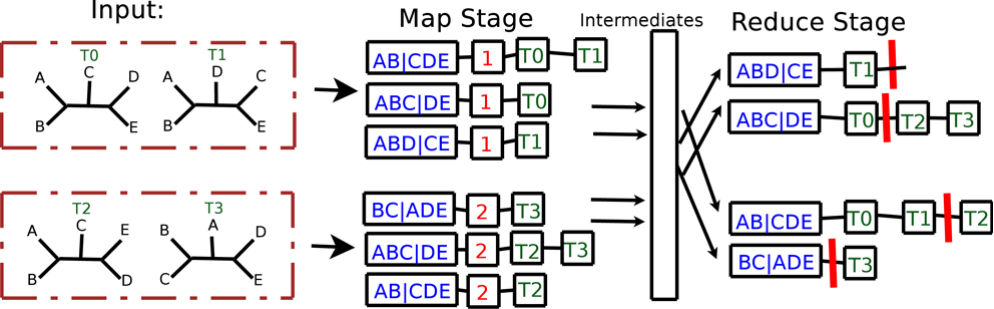
**Phase 1 of the MrsRF(*p*, *q*) algorithm**. Two mappers and two reducers are used to process the input files, where  = {*T*_0_, *T*_1_} and  = {*T*_2_, *T*_3_}.

In Figure 3, there are two input files, each containing two trees each. Trees in the first file are only compared to trees contained in the second file. In the figure, we assume there are only two mappers, where each mapper is responsible for handling one of the two input files. Each mapper creates a local hash table based on the trees that it receives by using a marker of "1" (for file 1) or "2" (for file 2) to keep track of trees and their bipartitions from the  and  sets, respectively. Each mapper then emits its marked hash table to the reduce stage. For example, in Figure 3, the first mapper emits the following (key, value) pairs: (*AB*|*CDE*, (1, *T*0, *T*1)), (*ABC*|*DE*, (1, *T*0)), and (*ABD*|*CE*, (1, *T*1)). These (key, value) pairs are processed in an intermediate stage, where each reducer processes all of the values associated with a particular key.

##### The first reduce stage

Once the map stage completes, each of the *r *reducers takes as input a (key, list(value)) pair with bipartition *B*_*i *_as the key and a list of tree id lists as the value. There will be at most *m *lists of tree ids for each bipartition. Each reducer then combines these *O*(*m*) lists in a manner such that the trees from file 1 are separated from the trees from file 2 to form a single line in the global hash table. Each row of the global hash table represents a unique bipartition among the *t *trees. Continuing with our example from Figure 3, the first reducer processes the lists associated with keys *ABD*|*CE*, and *ABC*|*DE*. Thus, the first reducer receives the list of lists (*ABD*|*CE*,(1, *T*1)) and (*ABC*|*DE*, (1, *T*0), (2, *T*2, *T*3)) and outputs the final (key, value) pairs of (*ABD*|*CE*, (*T*1||)), and (*ABC*|*DE*, (*T*0 ||*T*2, *T*3)), respectively. The symbol || denotes a partition that separates trees from the first input file with trees from the second input file.

#### Phase 2 of the MrsRF(*p*, *q*) algorithm

##### The second map stage

**Figure 4:**
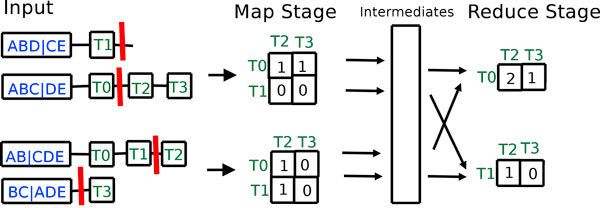
**Phase 2 of the MrsRF(*p*, *q*) algorithm**. Once again, there are two mappers and two reducers. The horizontal bars between elements represent a partition that separates trees from one file from trees in the other. Bipartitions containing elements on only one side of the partition are discarded.

Each of the *m *mappers receives an equal portion of the global hash table based on the total number of comparisons required to process the (key,value) pairs. In Figure 4, key (*ABC*|*DE*) has as its list of values (*T*0||*T*2, *T*3). Two total comparisons will be done since *T*0 is compared to *T*2 and *T*3. In general, if there are *u *tree ids on the left side of || and *v *trees on the right side, then *uv *total comparisons are required. Each mapper then computes a local similarity matrix from its portion of the hash table. In the second reduce stage, this similarity matrix will be converted into a RF (or dissimilarity) matrix.

Consider Figure 4. The first mapper has two rows of the global hash table assigned to it. Next, it computes a *p *× *q *similarity matrix from those hash table elements. In our example, the resulting similarity matrix is of size 2 × 2. To do this, it compares the tree ids in the first partition of a row to the tree ids located in the second partition of a row, and increments the local similarity matrix accordingly. For example, for the hash table row (*ABC*|*DE*, (*T*0 ||*T*2, *T*3)), the first mapper increments by one the locations (*T*0, *T*2) and (*T*0, *T*3) in its local similarity matrix. Rows that do not contain elements in both of its partitions are discarded. Therefore, in Figure 4, the hash table row (*ABD*|*CE*, (*T*1 ||)) is discarded. Each increment to entry (*i*, *j*) in the similarity matrix represents that the pair of trees *T*_*i *_and *T*_*j *_share a bipartition.

Once, a mapper has finished processing its hash table, it emits its similarity matrix for processing in the reduce stage. The key is the row id and the value is the contents of that row id. Thus, in Figure 4, the first mapper emits the (key, value) pairs (*T*0, (1, 1)), and (*T*1, (0, 0)).

##### The second reduce stage

Here, the input is a similarity matrix row identifier, *i*, and a list of rows that contain the local similarity scores found by each of the mappers. For a particular key, the number of rows within the list of rows received by a reducer is equal to the number of mappers, *m*. In Figure 4, the first reducer receives the following (key, value) pair for similarity identifier, *T*0: (*T*0, (1, 1), (1, 0)). The reducer sums up the columns of each of the lists to produce (*T*0, (2, 1)). To produce the RF distance for row *T*_*i*_, each column in the final similarity matrix is subtracted from *n *- 3, the maximum possible RF distance where *n *represents the number of taxa in the *t *trees of interest. Together, the output from the reduce stage yields a final submatrix. For each node *N*_*i*_, the resulting submatrix is written to a file. These files can then be combined to form a final RF matrix, or be kept in their partitioned form for easier handling.

### Analysis of MrsRF

MrsRF(*p*, *q*) is where all of the computation for the MrsRF algorithm lies. At least one node will require *O*(*t*) time to obtain the trees for its  and  sets. The first map phase of the MrsRF(*p*, *q*) algorithm, which is based on HashRF's first phase, requires , where *n *is the number of taxa and *m *is the number of mappers. *O*(*n*(*p *+ *q*)) is the total number of bipartitions that must be processed across the *p *+ *q *trees and inserted into the hash table. Suppose *b *unique bipartitions are found. In the worst case, a bipartition has a length of *p *+ *q*, which re ects the fact that it appears in all *p *+ *q *trees. Hence, the complexity is  for the first reduce phase, where *r *is the number of reducers. For the second phase of the MrsRF(*p*, *q*) algorithm, in the worst case, each mapper requires  to produce its local similarity matrix. Each reducer requires  time. Hence, if *p *and *q *are large enough, phase 2 is more time-consuming than phase 1 in the MrsRF algorithm. Our analysis does not incorporate communication costs as there is not an explicit model of communication for the MapReduce framework.

### Biological trees

Below, we describe the biological trees used in this study were obtained from two recent Bayesian analysis.

1. 20,000 trees obtained from a Bayesian analysis of an alignment of 150 taxa (23 desert taxa and 127 others from freshwater, marine, and oil habitats) with 1,651 aligned sites [13]. Two independent runs consisting of 25 million generations (with trees sampled every 1,000 generations) were performed with four independent chains in MrBayes using the GTR+I+Γ model.

2. 33,306 trees obtained from an analysis of a three-gene, 567 taxa (560 angiosperms, seven outgroups) dataset with 4,621 aligned characters, which is one of the largest Bayesian analysis done to date [14]. Twelve runs, each with four chains, ran for at least 10 million generations in MrBayes using the GTR+I+Γ model. Trees were sampled every 1,000 generations.

Table 1 presents statistical data on our collection of biological trees. Both collections contain unique trees as each tree appears once. The total number of bipartitions in a collection of *t *binary trees is *t*(*n *- 3), where *n *is the number of taxa. This is the number of bipartitions that must be processed by MrsRF in order to compute the *t *× *t *RF matrix. Many of the these bipartitions are shared across the trees. There are 1,168 and 2,444 unique bipartitions among the 150 and 567 taxa trees, respectively. The hash table size is the result of the first reduce stage of the MrsRF(*p*, *q*) algorithm. The RF matrix data is the output of the second reduce stage of the algorithm. When MrsRF is executing for speed in our experiments, the hash table from the first reduce stage of MrsRF(*p*, *q*) is kept in memory.

**Table 1:** Statistics for our Bayesian tree collections

number of taxa	total trees (*t*)	total bipartitions	unique bipartitions	hash table size	RF matrix cells (*t *× *t*)	RF matrix size
150	20,000	2.9 × 10^6^	1168	16 MB	4 × 10^8^	1.2 GB
567	33,306	18.8 × 10^6^	2444	102 MB	1.1 × 10^9^	3.3 GB

### Implementation and platform

All experiments were run on a multi-core cluster with configurations ranging from 1 to 32 nodes. Each node consists of a PowerEdge 1950 1U server, with two Intel Xeon E5420 2.5 GHz quad-core processors, resulting in a total of eight cores. Each node also consists of 16 GB DDR2 667 MHz fully-buffered DRAM and 160 GB of hard-disk. The nodes are connected together with a gigabit ethernet switch. We modified the Phoenix runtime system (Original Release version) to work on 64-bit Linux platforms, as the cluster runs the CentOS 5.2 64-bit operating system on all nodes. HashRF and HashRF(*p*, *q*) [11] are written in C++ and MrsRF and Phoenix are implemented in C. All programs are compiled with gcc 4.1.2 with the -03 compiler option.

## Results and discussion

### Establishing the fastest sequential algorithm

We evaluate the performance of MrsRF on our computational platform as we vary the number of cores, the number of nodes, and the problem size of interest. First, we establish the fastest sequential algorithm in order to compute the speedup of our approach. Speedup is defined as  where, *T*_1 _is the time required by the fastest sequential program and *T*_*N *× *c *_is the time required by MrsRF run on *N *nodes and *c *cores. Previous experiments established HashRF and HashRF(*p*, *q*) as the fastest sequential algorithms for computing the RF matrix [11,15].

**Figure 5:**
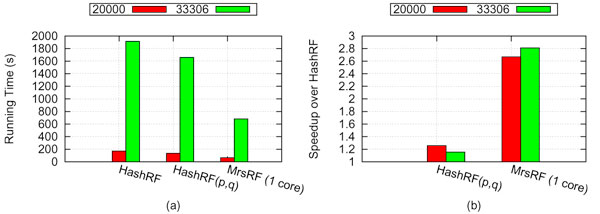
**Sequential running time and speedup of HashRF, HashRF(*p*, *q*), and MrsRF (1 core) algorithms on 20,000 and 33,306 trees on 150 and 567 taxa, respectively**. (a) denotes the running time for each algorithm. (b) denotes the speedup of HashRF(*p*, *q*) and MrsRF (1-core) over HashRF.

Figure 5 compares the sequential running time of HashRF, HashRF(*p*, *q*), and MrsRF. Each data point represents the average of five runs of the algorithm for each dataset. Surprisingly, our experiments showed that our MrsRF algorithm using 1 core is up to 2.8 times faster than HashRF on larger tree sets. This corresponds to an average time of 680.9 seconds for MrsRF compared to a running time of 1913.22 for HashRF, and a running time of 1657.75 for HashRF(*p*, *q*). The difference in performance is due to language-specific implementation decisions. MrsRF is written in C to match the implementation language of Phoenix. HashRF and HashRF(*p*, *q*), on the other hand, are C++ implementations that employ the Standard Template Library (STL) and classes, which introduces extra overhead when compared to MrsRF's ANSI C implementation. Thus, all our speedup results are in relation to MrsRF run on a single core.

### Multi-core performance of MrsRF

**Figure 6:**
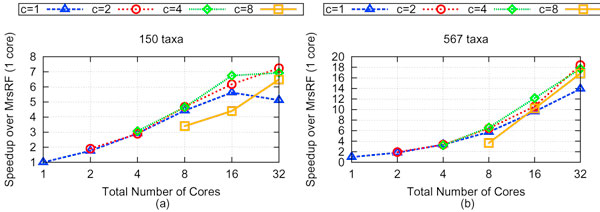
**Speedup of MrsRF algorithm on various *N *× *c *multi-core cluster configurations**. (a) shows the speedup of MrsRF on various *N *× *c *configurations over MrsRF (1-core) for 150 taxa and 20,000 trees. (b) shows the speedup of MrsRF on various *N *× *c *configurations over MrsRF (1-core) for 567 taxa and 33,306 trees.

Figure 6 shows the speedup of MrsRF on our 20,000 and 33,306 tree sets over the total number of CPUs utilized, ranging from 1 to 32 cores. Every dataset was run five times using various cluster configurations. The numbers reported are the average between each set of five runs. Speedup is calculated with respect to MrsRF run serially on one core.

#### Cluster configurations

To test how much a factor architecture is to speedup, we used different system configurations to measure performance. Let *N *denote the number of nodes used, and *c *denote the number of cores used on each node. For any *N *× *c *configuration, there are *Nc *total cores being utilized. Thus, for 8 total cores to be used, we run our algorithm using 1 × 8, 2 × 4, 4 × 2 and 8 × 1 configurations. Each curve denotes the number of cores utilized per node. Therefore, if *c *= 4 and the total number of cores is 8, then this data point reflects a 2 × 4 configuration. Likewise, if *c *= 1 and the total number of cores is 32, then the data point reflects a 32 × 1 configuration.

#### 150 taxa trees

Figure 6 shows that as the number of bipartitions increase, so does the performance of MrsRF. While the curves for *c *= 2 and *c *= 4 performs the best, the *c *= 1 and *c *= 8 configurations performs the poorest. Performance differences across various cluster configurations are underscored as the total number of cores increases. This is due to overhead in partitioning the data (*c *= 1) and inter-node resource contention for memory bandwidth (*c *= 8). Despite this, an increase in total cores results in an increase in performance, and we see our best performance when 16 total nodes is utilized, with a maximum speedup of 7.25 for this dataset. This corresponds to an average running time of 8.73 seconds for MrsRF on 32 cores.

#### 567 taxa trees

**Figure 7:**
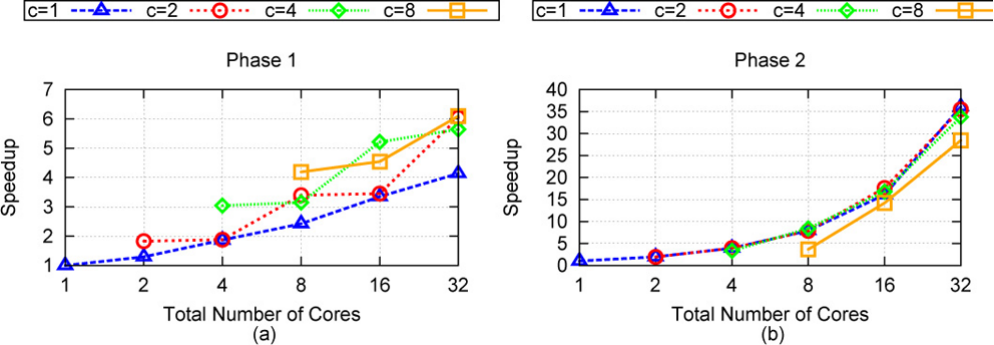
**Performance of Phase 1 and Phase 2 of MrsRF(*p*, *q*) on 567 taxa and 33,306 trees**. Here, the 567 taxa - 33,306 tree set is of interest. (a) shows the speedup of Phase 1 of the MrsRF algorithm run on different *N *× *c *configurations over Phase 1 of MrsRF (1-core). (b) shows the speedup of Phase 2 of the MrsRF algorithm run on different *N *× *c *configurations over Phase 2 of MrsRF (1-core).

We see a similar trend in architectural performance in the 567 taxa case, thus underscoring the importance of managing resource contention and communication overhead in relation to performance. However, with the increased bipartitions present in the 567 taxa case, we see an markedly increased amount of speedup, with a maximum amount of speedup of 18.4 attainable with 32 cores using a 16 × 2 cluster configuration. The maximum speedup corresponds to an average running time of 36.93 seconds. In comparison, it took the serial execution of MrsRF an average of 680.9 seconds to compute the RF matrix, while it took HashRF and HashRF(*p*, *q*) an average of 1913.22 and 1657.75 seconds respectively. Our results show that MrsRF is a very scalable approach for computing the all-to-all RF Matrix, with performance increasing with large problem sizes. Figure 7 shows that Phase 2 of the MrsRF(*p*, *q*) approach exhibits linear speedup. Overall speedup of MrsRF increases (decreases) when Phase 2 (Phase 1) of MrsRF(*p*, *q*) dominates the computation time. Once again, the differences in speedup that we observe with different *N *× *c *configurations suggest that multiple cluster configurations should be run to achieve the maximum speedup.

### RF matrix application: Visually summarizing tree collections

**Figure 8:**
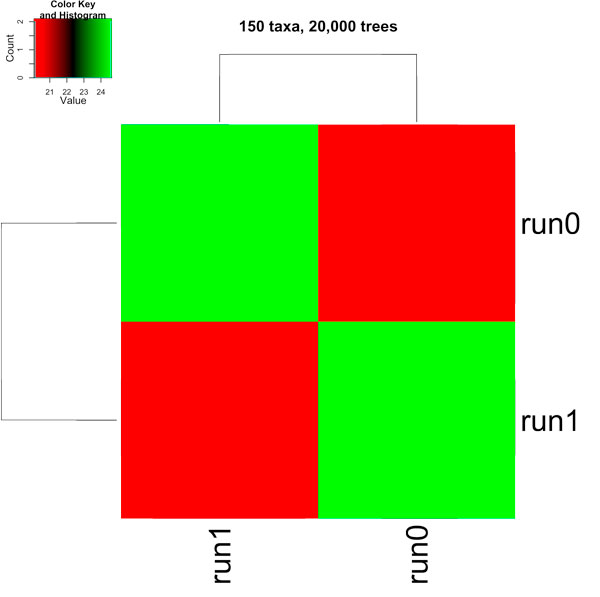
**A heatmap illustrating the clustering of the biological trees across MrBayes runs for the 150 taxa and 20,000 tree set**.

**Figure 9:**
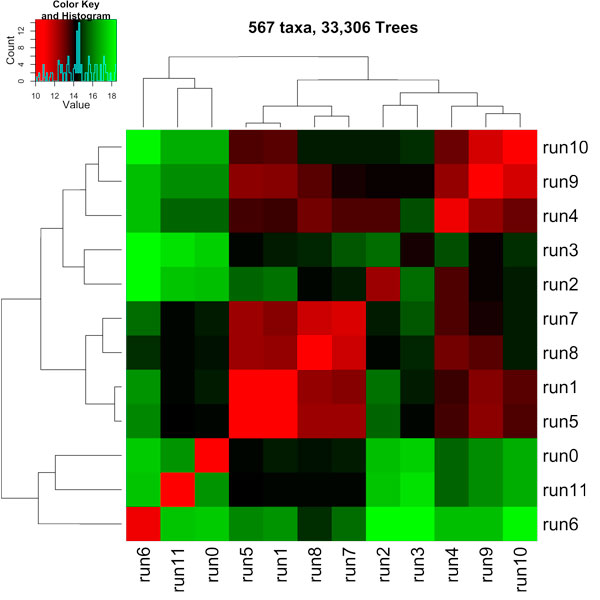
**A heatmap illustrating the clustering of the biological trees across MrBayes runs for the 567 taxa and 33,306 tree set**.

The fundamental question we address here is "what do the gathered trees tell us about the Bayesian analyses that produced them?" To answer this, we partitioned our *t *× *t *RF matrix based on the MrBayes run that generated the tree. Figure 8 shows a heatmap of our 20, 000 × 20, 000 RF matrix broken up into a 2 × 2 matrix, where each entry (*i*, *j*) shows the average RF rate between the trees from run *i *and run *j *of MrBayes. For this dataset, two MrBayes runs were used to create the entire collection of 20, 000 trees, where each run consisted of 10, 000 trees. Figure 9 shows the 33, 306 × 33, 306 RF matrix broken up into a 12 × 12 matrix. For this dataset, twelve MrBayes runs were used to create the entire collection of 33, 306 trees, where each run consisted of 2, 000 to 3, 000 trees.

In Figures 8 and 9, heatmap cell (*i*, *j*) is colored according to how similar (lower RF rates) the trees are across runs *i *and *j*. Hot regions, colored in shades of red, denote highly similar trees. Cool regions, colored in shades of green, denote dissimilar trees. Cell (run1, run1) in Figure 8 shows an average RF rate of about 20% while (run1, run2) show an average RF rate of around 24%. In Figure 9, cell (run6, run10) shows an average RF rate of about 18% while these runs compared to themselves (i.e., cells (run6, run6) and cells (run10, run10)) show higher levels of similarity with an average RF rate of around 11%. Finally, the histogram in the color key represent the number of cells with a particular RF value.

Hierarchical clustering, using the hclust function in R, is used to cluster highly similar cells in the heatmap to each other. For the 150 taxa tree collection, the trees from one run are not similar to trees in the other run suggesting that two different summaries are required to encapsulate the evolutionary relationships among the trees (see Figure 8). For the 567 taxa trees, the heatmap (in Figure 9) shows regions of high similarity among the trees within a run, and regions of dissimilarity across runs. The clustering also shows that runs 0, 6, 10 exhibit trees with high levels of similarity among them. One conclusion is that these runs converged to similar areas of tree space in the phylogenetic search and the trees from those runs can be summarized by a single tree (such as a consensus tree). This is also true for runs 1, 5, 7, and 8. The clustering of the other runs (runs 2 and 3) and (runs 0, 6, 11) have lower levels of similarity.

Overall, the clusterings in Figures 8 and 9 suggest that there exist several well-supported partitions of the trees and that each partition should be summarized separately in order to minimize information loss. Moreover, the data suggests that the various Bayesian runs among the 150 and 567 taxa trees did not converge to the same place in tree space. One of the greatest benefits of convergence is reliability of the trees found by a phylogenetic heuristic. Hence, RF matrices could be used as a method to detect convergence between runs.

## Conclusion

In this paper, we evaluate the applicability of the MapReduce framework for developing multi-core phylogenetic applications. We design a new algorithm called MrsRF for computing the all-to-all RF matrix using the MapReduce framework. An open-source implementation of our MrsRF algorithm is available from the web [9]. One of the novelties of computing an RF matrix in a MapReduce context is that the size of the input (*t *evolutionary trees) is much smaller than the size of the output (*t *× *t *RF matrix). Our results show that we achieve a significant speedup using MrsRF over the fastest sequential algorithm. On our largest problem size (567 taxa and 33,306 trees), we attain a maximum speedup of 18.4 on 32 cores. Our results suggest that MrsRF is a very scalable approach with increased performance resulting from larger collections of trees. Furthermore, our results show the usefulness of running an algorithm on multiple *N *× *c *multi-core cluster configurations to ensure that the best performance is attained. Finally, we show an application for RF matrices related to summarizing a collection of trees and detecting convergence of a phylogenetic analysis.

Additionally, our results suggest that the storing the hash table is a better alternative to storing the large RF matrices, which are not sparse. Since the hash table contains a list of shared, unique bipartitions, it is much smaller than the final *t *× *t *RF matrix. For example, storing the hash table of the 33, 306 × 33, 306 RF matrix takes only 102 MB of storage compared to the 3.3 GB necessary to store the full matrix (see Table 1). Given the speed of the MrsRF(*p*, *q*), especially in Phase 2, one could store the hash table and compute the resulting *t *× *t *RF matrix on the fly as needed.

Overall, our results show that MapReduce is an exciting approach for developing multi-core phylogenetic applications. Future work includes studying the performance of MrsRF on larger clusters and tree collections. Finally, we intend to design additional MapReduce phylogenetic applications—especially as it relates to reconstructing more accurate phylogenetic trees efficiently.

## Competing interests

The authors declare that they have no competing interests.

## Authors' contributions

SM and TW both designed and implemented the MrsRF algorithm. SM created all of the figures. Both authors contributed to writing the manuscript and have approved its final contents.
